# Feeding a Modified Fish Diet to Bottlenose Dolphins Leads to an Increase in Serum Adiponectin and Sphingolipids

**DOI:** 10.3389/fendo.2016.00033

**Published:** 2016-04-21

**Authors:** Philip M. Sobolesky, Tyler S. Harrell, Celeste Parry, Stephanie Venn-Watson, Michael G. Janech

**Affiliations:** ^1^Department of Medicine, Division of Nephrology, Medical University of South Carolina, Charleston, SC, USA; ^2^Grice Marine Laboratory, Department of Biology, College of Charleston, Charleston, SC, USA; ^3^Translational Medicine and Research Program, National Marine Mammal Foundation, San Diego, CA, USA

**Keywords:** parallel reaction monitoring mass spectrometry, FGF21, sphingosine-1-phosphate, insulin sensitivity, C17:0, proteomics, metabolic syndrome, ceramides

## Abstract

Feeding a modified fish diet has been suggested to improve insulin sensitivity in bottlenose dolphins; however, insulin sensitivity was not directly measured. Since demonstrating an improvement in insulin sensitivity is technically difficult in dolphins, we postulated that directional changes in the hormone axis: fibroblast growth factor 21 (FGF21)/Adiponectin/Ceramide (Cer), could provide further support to this hypothesis. We measured 2-h post-prandial serum FGF21, total adiponectin, percent unmodified adiponectin, ceramide, and sphingosine levels from dolphins fed a diet rich in heptadecanoic acid (C17:0) over 24 weeks. Serum FGF21 was quantified by ELISA with an observed range of 129–1599 pg/ml, but did not significantly change over the 24-week study period. Total adiponectin levels (mean ± SD) significantly increased from 776 ± 400 pmol/ml at week 0 to 1196 ± 467 pmol/ml at week 24. The percent unmodified adiponectin levels (mean ± SD) decreased from 23.8 ± 6.0% at week 0 to 15.2 ± 5.2% at week 24. Interestingly, although FGF21 levels did not change, there was a good correlation between FGF21 and total adiponectin (ρ = 0.788, *P* < 0.001). We quantified the abundances of serum ceramides and sphingosines (SPH) because adiponectin has a defined role in sphingolipid metabolism through adiponectin receptor-mediated activation of ceramidases. The most abundant ceramide in dolphin sera was Cer 24:1 comprising 49% of the ceramides measured. Significant reductions were observed in the unsaturated Cer 18:1, Cer 20:1, and Cer 24:1, whereas significant increases were observed in saturated Cer 22:0, Cer 24:0, and Cer 26:0. However, total serum ceramides did not change. Significant elevations were detected for total sphingosine, dihydrosphingosine, sphingosine-1-phosphate, and dihydrosphingosine-1-phosphate. Proteomic analysis of the serum proteins revealed few changes in serum proteins over the study period. In conclusion, shifting the dolphin diet to fishes rich in odd chain saturated fatty acids, such as C17:0, resulted in increased serum levels of the insulin sensitizing hormone adiponectin and serum SPH consistent with an insulin-sensitizing phenotype. It is still unclear whether FGF21 plays a role in the regulation of adiponectin in dolphins, similar to that shown in laboratory animal models.

## Introduction

Dolphins can develop a condition similar to humans with metabolic syndrome that includes elevated serum insulin, glucose, triglycerides, cholesterol, iron, saturated transferrin, ferritin, and total iron, with associated steatosis and iron overload (1–3). A study investigating differences in serum lipid profiles between two groups of dolphins, with high or low insulin, found differences in phospholipid fatty acids that may play a role in the susceptibility to or development of an insulin-resistant-like state (3). Higher blood levels of the fatty acids C17:0 (heptadecanoic acid), C20:4n6 (arachidonic acid), and C22:0 (behenic acid), in particular, were associated with lower insulin levels. A recently published 24-week pilot study investigated the effect of modifying the diet with species of fish that are rich in C17:0 (pinfish and mullet) in six dolphins. The investigators reported an elevation in serum C17:0 over the course of the study that was correlated with a significant reduction in serum ferritin levels. Furthermore, a marked reduction in post-prandial variability was noted for serum insulin, triglycerides, and glucose concentrations (3). This “correction” was interpreted as a possible increase in insulin sensitivity. Insulin sensitivity as determine by HOMA-IR scoring has not been developed for dolphins and the use of a hyperinsulinemic–euglycemic clamp to define insulin resistance in these animals is very invasive and not reasonable given the protections afforded these animals. Therefore, we postulated that directional changes in other hormone systems, known to improve insulin sensitivity, should also change in a direction that supports the hypothesis of improving insulin sensitivity in this small pilot cohort.

Insulin sensitivity is controlled through a complex interaction of multiple hormones and target tissues (4). One of the more prominent systems is the fibroblast growth factor 21 (FGF21)/Adiponectin/Ceramide axis (5). FGF21 is a potent regulator of metabolism and energy utilization by exerting its glycemic and insulin-sensitizing effects through the upregulation of adiponectin secretion (5). Adiponectin is an adipokine that forms oligomers and circulates as trimers, hexamers, and high molecular weight (HMW) forms (6). The relevant action of each oligomer is tissue specific, with the HMW form known to exert a greater response on hepatocytes, thereby modulating gluconeogenesis, lipolysis, and insulin sensitivity (7, 8). Increased adiponectin levels have been associated with better glycemic control and lipid profiles in humans with type 2 diabetes (9, 10). In healthy laboratory animals, adiponectin signaling increases ceramidase activity that converts ceramides into sphingosines (SPH) (11). Ceramides are lipid metabolites that promote apoptosis, inflammation, and insulin resistance, whereas SPH promote cell survival and anti-inflammatory actions (12). Imbalance in the ceramide and sphingosine rheostat has been associated with obesity and insulin resistance (13, 14).

The HMW adiponectin protein forms as a result of glycosylation of lysine residues in its collagenous domain (15). The concentration of HMW adiponectin, and not total adiponectin levels, has been shown to improve insulin sensitivity (16). Our previous work validated a parallel reaction monitoring mass spectrometry (PRM-MS) assay for dolphin total adiponectin and described a second fractional peptide measurement at lysine 75 (% unmodified) whose presence implicated this measurement as a proxy of HMW adiponectin (17). The proportion of this unmodified at lysine 75 peptide to total adiponectin was able to differentiate between dolphins with hemochromatosis and elevated post-prandial insulin levels versus healthy dolphins (17).

For the dolphins in the 24-week pilot study (3), we hypothesized that these animals (1) will have an intact FGF21/Adiponectin/Ceramide axis similar to other mammals and (2) will increase in serum FGF21, adiponectin, and sphingosine and will decrease in serum percent unmodified adiponectin and ceramides in response to the modified fish diet in dolphins. To test our hypothesis, we quantified the serum levels of FGF21, adiponectin, percent unmodified adiponectin, ceramides, and SPH in the same six bottlenose dolphins on a modified diet as reported in Venn-Watson et al. (3). Furthermore, we investigated whether these measurements correlated with blood laboratory measurements used to characterize metabolic syndrome. Information-dependent acquisition-based LC-MS/MS proteomics was also used to investigate changes in non-target, highly abundant, serum proteins.

## Materials and Methods

### Study Population

The bottlenose dolphin (*Tursiops truncatus*) modified diet study population was utilized in this study and has been previously described (3). The study was conducted under the Marine Mammal Program (MMP) IACUC-approved animal care and use protocol #101–2012. Animals E, W, and Q were females ages 12, 51, and 32 years, respectively. Animals V, A, and BB were males ages 42, 35, and 8 years, respectively. Briefly, six dolphins had a baseline diet of 70–80% capelin and 20–30% mix of herring, mackerel, and/or squid that was modified to 25% capelin, 25% mullet, and/or pinfish, and 50% mix of herring, mackerel, and/or squid. Total daily kilocalories remained the same. The modified diet resulted in significantly increased daily intake of C17:0. Proximate analyses for fish nutrients (kilocalories, % moisture, % protein, % fat, % ash, and % carbohydrates) are routinely measured by Michelson Laboratories, Inc. (Commerce, CA, USA) for every fish lot that is part of Navy marine mammal diets. Thus, nutrient data for the baseline and modified diets for our study were available and can be found in Table S1 in Supplementary Material. Daily dietary kilocalories were calculated per kilogram of body weight for each dolphin. Daily moisture, protein, fat, ash, and carbohydrate intake was calculated as total grams ingested per kilogram of body weight for each dolphin. Daily nutrient intake was then compared between baseline and modified diets using a Wilcoxon two-sample test. There were no significant differences in total daily dietary kilocalories, moisture, protein, fat, ash, or carbohydrate when comparing baseline and modified diets.

### Samples

Aliquots of dolphin serum were shipped overnight on dry ice from the feeding study population of six bottlenose dolphins in the U.S. Navy Marine Mammal Program, as previously reported (3). Upon arrival, the samples were flash thawed in a 40°C water bath for 2 min, vortexed for 10 s, and centrifuged at 3000 × *g* for 10 s in a tabletop centrifuge before being aliquoted into 110 and 50 μl aliquots that were then frozen at −80°C until tested.

### Fibroblast Growth Factor 21

Serum FGF21 concentrations were determined using the Fibroblast Growth Factor 21 Mouse/Rat ELISA kit (Biovendor, Asheville, NC, USA) guided by the manufacturer’s instructions. A detailed description can be found in Data Sheet 1 in the Supplemental Data section. Batch corrections were not applied as the inter-assay coefficient of variation for the mean of the reference material was 0.23%. Intra-assay coefficient of variation was 8.2%.

### Ceramides

Serum aliquots (110 μl) were submitted to the MUSC Lipidomics Core for ceramide and sphingosine determination following the established protocol of Ref. (18). Briefly, serum was diluted in serum-free media and spiked with internal standard solutions to quantify the following: SPH, dihydro-sphingosines (dSPH), sphingosine-1-phosphates (S1P), dihydro-sphingosine-1-phosphates (dS1P), Ceramides (Cer 16:0, Cer 14:0, Cer 16:0, Cer 18:0, Cer 18:1, Cer 20:0, Cer 24:0, Cer 24:1, Cer 26:0, and Cer 26:1), and dihydro-ceramide (Cer d16:0). The ceramides quantified contained a d18:1 sphingoid backbone and the numbers refer to the number of carbons:number of double bonds in the N-linked fatty acid. Lipids were extracted using a solution of 30:10:60 isopropanol:water:ethyl acetate. Samples were vortexed, and centrifuged at 4000 × *g* for 10 min. Supernatant was transferred to a new tube, formic acid was added, and the extraction process was repeated. Supernatants were then combined, evaporated, and reconstituted in mobile phase A (1 mM ammonium formate in methanol containing 0.2% formic acid). This was vortexed and centrifuged for 5 min at 4000 × *g*. Samples were analyzed on a triple quadrupole mass spectrometer equipped with electrospray ion source (Thermo Finnigan). Concentrations were determined by external standard curve. Any sample that did not exceed the concentration of the blank by a factor of two was considered below limit of detection. Data are reported as picomole per milliliter.

### Reagents, Chemicals, and Synthetic Peptide Standards

Reagents used were ACS grade or better. Water, acetonitrile, and methanol were LC-MS grade (Honeywell Burdick & Jackson, Morristown, NJ, USA). Synthetic stable isotope-labeled peptides for adiponectin were previously described in detail (17) and were synthesized by New England Peptide (Gardner, MA, USA).

### Preparation of Tryptic Peptides

The preparation of serum for analysis of adiponectin was performed following an established protocol (17) with the following modifications. An aliquot of dolphin serum was thawed at room temperature for 1 min, vortexed for 5 s, then diluted (1:10; v:v) in 50 mM ammonium bicarbonate (AmBic). A solution of dithiothreitol (dissolved in 25 mM AmBic) was mixed by pipet to a final concentration of 100 mM, then centrifuged briefly, and incubated at 60°C for 30 min. The reaction was allowed to cool for 5 min, then alkylated by the addition of iodoacetamide (dissolved in 50 mM AmBic) to a final concentration of 10 mM, and incubated at 37°C for 30 min. The reaction was diluted with 176.5 μl 50 mM AmBic before adding mass spectrometry grade trypsin gold (Promega, Madison, WI, USA) at a 1:10 ratio of enzyme to protein. The reaction was incubated at 37°C for 16 h, then stopped by the addition of 350 μl of 1% formic acid, and incubated at room temperature for 30 min. The two isotopically labeled standards were added to each sample, completed to 1 ml with 0.1% formic acid, and then loaded the sample onto an acetonitrile conditioned Strata-X 33 μ polymeric reverse phase solid phase extraction column (Phenomenex, Torrance, CA, USA). The column was washed twice with 1 ml 0.1% formic acid. Peptides were eluted first with 1 ml of 15% acetonitrile/0.1% formic acid then in a separate tube with 1 ml 30% acetonitrile/0.1% formic acid. Eluted samples were frozen at −80°C overnight, then dried down under vacuum by speedvac. Each sample was resuspended in 100 μl MPA (98% water, 2% acetonitrile, 0.1% formic acid), vortexed for 15 min, then centrifuged at 10,000 × *g* for 5 min before being transferred to a new 1.5 ml microcentrifuge tube. Peptide concentration was estimated by absorbance at 280 nm (average ~14 μg/μl). Prior to injection, 5 μl of the sample was diluted into 195 μl of MPA, and then injected onto the trap column.

### Adiponectin Quantification

Peptides of total and Lys-75 unmodified adiponectin were quantified using previously published protocols from Neely et al. (17) with the following modifications. Tryptic peptides (10 μl) were loaded onto a 100 μm × 2 cm C18 (100 Å with 5 μm particles) trap column (Acclaim PepMap^®^ 100; Thermo Fisher Scientific) and separated on a 75 μm × 15 cm C18 (100 Å with 3 μm particles) analytical column (Acclaim PepMap100^®^; Thermo Fisher Scientific). Reverse phase separation occurred at 350 nl/min on a 2D+ NanoLC Ultra system (Eksigent, Dublin, CA, USA). The LC was connected *via* nanospray source to a Triple-TOF 5600 mass spectrometer (AB Sciex, Foster City, CA, USA).

Dolphin sera were processed in randomized batches of 9. One serum in each batch was processed in triplicate to determine experimental variability. One serum in each batch was injected in triplicate to account for intra-batch variability. Each batch also contained a standard reference material (SRM) serum to correct between experimental batches, and an experimental blank consisting of phosphate-buffered saline (PBS). The blank was processed identically to serums in each batch and contained the same amount of trypsin as the SRM. For PRM experiments, the instrument was set in positive ion mode and TOF-MS data were collected in a window of 450–1250 *m/z* for 150 ms, followed by each parent ion MS/MS for 200 ms, from 100 to 1600 *m/z*. Total dolphin adiponectin (IFY) was quantified by comparing the ratio between the native IFY y13^2+^ product ion (586.9^3+^ → 749.83^2+^ *m/z*) and the corresponding product ion from the SIS peptide (589.6^3+^ → 753.83^2+^ *m/z*). The amount of Lys-75 unmodified (GDT) dolphin adiponectin was quantified by comparing the ratio between the native GDT y7^+^ product ion (716.34^2+^ → 715.37^+^ *m/z*) and the corresponding product ion from the standard peptide (721.34^2+^ → 725.37^+^ *m/z*). The amount of percent unmodified was calculated as (GDT/IFY) × 100.

### Proteomic Analysis of Serum

One microliter of dilute peptides (1:40; v/v, ~1 μg) was loaded onto the trap at 5 μl/min for 5 min before reverse phase separation at 350 nl/min from 0 to 40% mobile phase B (95% acetonitrile in 0.1% formic acid) over 50 min. Tryptic peptides were identified by performing runs in positive ion, information-dependent acquisition mode with product ion scans for 50 ms with up to 20 product ion scans if precursors were 350–1250 *m/z*, exceeded 100 cps, and had a 2+ to 5+ charge state (AnalystTF1.6). Raw data files generated by the AB Sciex 5600 were converted to a peak list using the AB Sciex MS Data Converter (v. 1.3. beta, June 2012). Protein identifications were made using Mascot Daemon (v. 2.4.0) searching against the Ensembl (release 64) turTru1 dolphin genome assembly protein database [16,599 sequences; Lindblad-Toh (19)] and the common Repository of Adventitious proteins database (cRAP; 2012.01.01; the Global Proteome Machine) using the following parameters: trypsin was selected as the enzyme and two missed cleavages were allowed; carbamidomethylation (Cys) was specified as a fixed modification; Gln → pyro-Glu (N-term Q) and Oxidation (M) were specified as variable modifications; a peptide tolerance of 20 ppm and MS/MS tolerance of 0.1 Da; instrument type was set to ESI-QUAD-TOF. Mascot files were then uploaded into Scaffold Q+ (v.4.4.5) for analysis with a protein threshold set to 1.0% false discovery rate, a minimum number of peptides set to 3, and a peptide threshold set to 50%. Proteins were excluded from analysis that did not have a spectral count >10 in at least one time point, or were missing from two or more dolphin samples at a time point. The quantitative value was normalized to total TIC with a normalization value set to 0. Values were imported into sigma plot 11.0 and log10 transformed to improve normality of the data. Proteomics data have been deposited to the ProteomeXchange Consortium (http://proteomecentral.proteomexchange.org) *via* the PRIDE partner repository (20) with the dataset identifier PXD003425.

### Statistics

All statistical analyses were performed with SigmaPlot 11.0 (Systat Software). Pearson Product Moment Correlation analysis was performed to detect correlations between adiponectin, FGF21, ceramides, SPH, and previously measured serum components, i.e., insulin, glucose, tryglycerides, iron, transferrin saturation, ferritin, ceruloplasmin, and haptoglobin (3). Adiponectin, ceramide, and sphingosine time-series data were analyzed by one-way ANOVA for repeated measures. For data that did not pass equal variance or normality tests, data were log transformed and the ANOVA was repeated. For the proteomics data, a repeated one-way ANOVA was performed on the log-transformed values that met the inclusion criteria. *Post hoc* comparisons versus time 0 were made using the Holm–Sidak *post hoc* test. Differences were considered statistically significant if *P* < 0.05. Fold change for each identified protein was calculated by dividing the mean quantitative value at a time point by the mean quantitative value at time 0.

## Results

### Modified Diet Is Associated with Increased Levels of Total Adiponectin and Reduced Levels of Percent Unmodified Adiponectin

**Figure 1:**
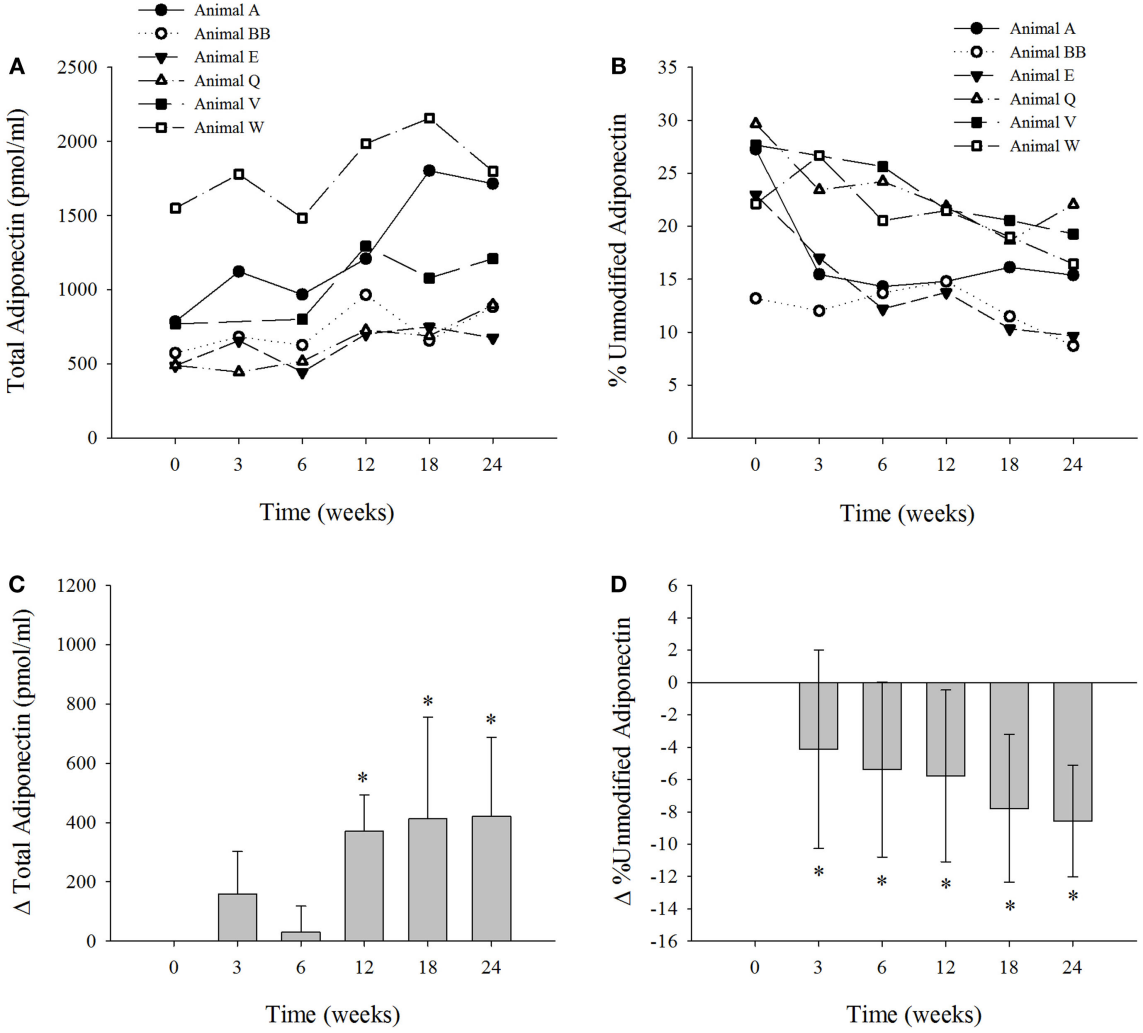
**The six bottlenose dolphins on a modified fish diet display an increase in total serum adiponectin and a decrease in percent unmodified adiponectin**. Symbols and lines correspond to the measured values of **(A)** total adiponectin (picomole per milliliter) and **(B)** percent unmodified adiponectin in each dolphin from time 0 to week 24. **(C)** The mean change in total adiponectin and **(D)** mean percent change in percent unmodified adiponectin for the six dolphins at each time point over the 24-week study (*n* = 6; except for week 3, *n* = 5). Data are presented as means and error bars indicate SD. A repeated measures one-way ANOVA with a Holm–Sidak *post hoc* test was used to determine significant changes in adiponectin levels versus week 0 (*denotes *P* < 0.05).

We measured the levels of total and percent unmodified adiponectin (Figures 1A,B) in six dolphins on the modified diet (3). Adiponectin levels were within previously reported ranges (17). The mean levels of adiponectin (pmol/mL ± SD) for the dolphins at 0-weeks was 776 ± 401; 3 weeks, 937 ± 531; 6 weeks, 806 ± 382; 12 weeks, 1147 ± 477; 18 weeks, 1189 ± 640; and 24 weeks, 1196 ± 467. The change in serum adiponectin levels were significantly elevated in the dolphins at weeks 12, 18, and 24 (*P* < 0.002) compared to week 0 (Figure 1C). The mean levels of percent unmodified adiponectin (mean% unmodified ± SD) in the dolphins at week 0 was 23.8 ± 6; 3 weeks, 18.9 ± 6; 6 weeks, 18.4 ± 6; 12 weeks, 18.0 ± 4; 18 weeks, 16.0 ± 4; 24 weeks, 15.2 ± 5. The mean change in percent unmodified adiponectin was reduced (*P* < 0.03) at all collection intervals versus control at week 0 (Figure 1D). Only one dolphin contained serum FGF21 concentrations below the LLOQ. In the other five dolphin samples above the LLOQ, serum FGF21 concentrations ranged from 129 to 1599 pg/ml. The mean change in FGF21 levels was not significantly different over the course of the study (Figure S1 in Supplementary Material). Previously published serum insulin levels for each dolphin (3) are presented in Figure S2 in Supplementary Material.

### Associations of Adiponectin with Metabolic Variables

Blood laboratory values were previously measured (3) and the values were used to calculate Pearson product moment correlations with serum adiponectin, percent unmodified adiponectin, and FGF21 levels. Serum adiponectin was positively associated with FGF21 (ρ = 0.788, *P* < 0.001) and heptadecanoic acid C17:0 (ρ = 0.441, *P* = 0.008) and negatively associated with ferritin (ρ = −0.425, *P* = 0.011), transferrin saturation (ρ = −0.381, *P* = 0.024), and iron (ρ = −0.433, *P* = 0.009) (Table 1). The amount of percent unmodified adiponectin was negatively correlated with total SPH (ρ = −0.434, *P* = 0.009) and positively correlated with insulin (ρ = 0.425, *P* = 0.011) and ferritin (ρ = 0.422, *P* = 0.012) (Table 1). FGF21 was negatively correlated with iron (ρ = −0.430, *P* = 0.013) (Table 1). Adiponectin and FGF21 were both negatively correlated with Cer 14:0, Cer 18:0, Cer 18:1, Cer 20:1, and Cer 22:1 (Table 1). No significant correlations were observed between the levels of serum adiponectin, percent unmodified adiponectin, or FGF21 with total ceramides, glucose, triglycerides, ceruloplasmin, and haptoglobin (Table S2 in Supplementary Material).

**Table 1:** **Significant Pearson product moment correlations (ρ) for adiponectin, percent unmodified adiponectin, and FGF21 with ceramides and blood laboratory measurements**.

Measured metabolic variables	Adiponectin^a^ (ρ)	*P*-value	% Unmodified adiponectin^a^ (ρ)	*P*-value	FGF21^b^ (ρ)	*P*-value
Cer 14:0	−**0.375**	**0.026**	0.018	0.921	−**0.457**	**0.008**
Cer d16:0	−0.099	0.569	**0.380**	**0.024**	−0.001	0.997
Cer 18:0	−**0.456**	**0.006**	−**0.439**	**0.008**	−**0.412**	**0.017**
Cer 18:1	−**0.490**	**0.003**	0.150	0.390	−**0.384**	**0.027**
Cer 20:0	−0.276	0.108	−**0.495**	**0.002**	−0.179	0.319
Cer 20:1	−**0.650**	**<0.001**	−**0.354**	**0.037**	−**0.522**	**0.002**
Cer 22:1	−**0.615**	**<0.001**	−0.029	0.867	−**0.482**	**0.005**
dSPH	−0.089	0.609	−**0.404**	**0.016**	−0.286	0.107
S1P	0.143	0.412	−**0.391**	**0.020**	−0.099	0.583
Total sphingosines	0.075	0.669	−**0.434**	**0.009**	−0.23	0.198
FGF21	**0.788**	**<0.001**	0.202	0.261	–	–

Venn-Watson et al. (3) Values						
Insulin	0.120	0.493	**0.425**	**0.010**	0.072	0.692
Iron	−**0.433**	**0.009**	−0.053	0.763	−**0.430**	**0.013**
Transferrin saturation	−**0.381**	**0.024**	−0.221	0.202	−0.344	0.050
Ferritin	−**0.425**	**0.011**	**0.422**	**0.012**	−0.306	0.083
Heptadecanoic acid (C17:0)	**0.441**	**0.008**	0.161	0.357	0.218	0.223

*^a^*n* = 35*.

*^b^*n* = 33*.

*Significance *P* < 0.05 are indicated in bold*.

### Significant Changes in Ceramide Levels Were Observed with a Modified Diet

**Figure 2:**
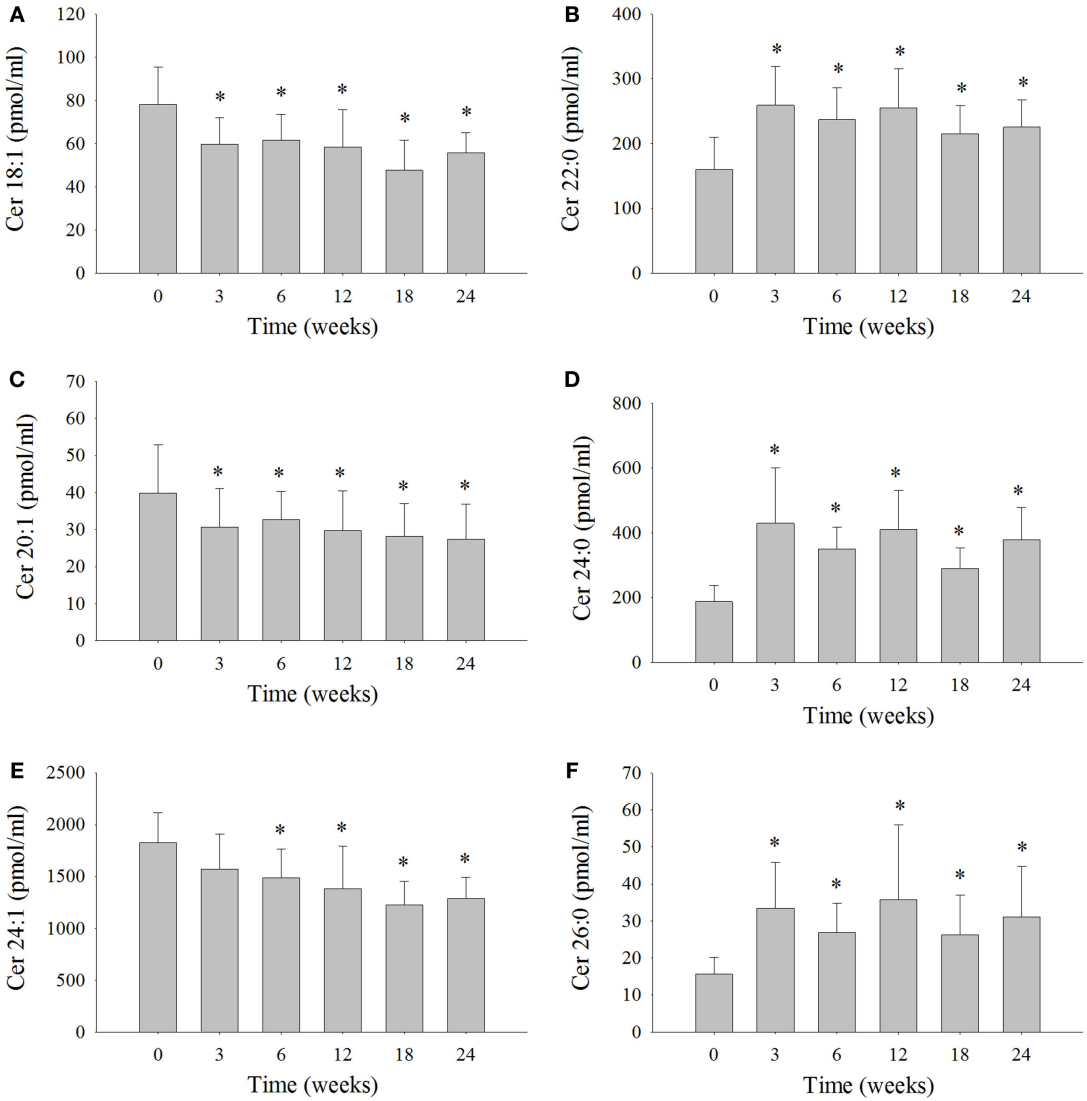
**Serum ceramide levels that significantly changed over the 24-week study in bottlenose dolphins on a modified fish diet**. Ceramides **(A)** Cer 18:1, **(B)** Cer 20:1, **(C)** Cer 22:0, **(D)** Cer 24:0, **(E)** Cer 24:1, and **(F)** Cer 26:0 were measured by LC/MS. A repeated measures one-way ANOVA with a Holm–Sidak *post hoc* test was used to determine significant changes in individual ceramide levels versus week 0 (* denotes *P* < 0.05).

Serum ceramide levels were measured in the six dolphins at each time interval (Figure 2; Table S3 in Supplementary Material). Ceramide 24:1 was the most abundant ceramide measured in the serum of the six dolphins comprising 40% on average of the total ceramides measured (Table S3 in Supplementary Material). The levels of Cer 24:1 were significantly reduced 18% at week 6, 24% at week 12, 33% at week 18, and 29% at week 24 compared to week 0 (Figure 2E). Ceramide 18:1 comprised about 2% of the total ceramides measured and was reduced 18% at week 3, 21% at week 6, 24% at week 12, 39% at week 18, and 27% at week 24 compared to week 0 (Figure 2A). Ceramide 20:1 comprised ~1% of the total ceramides measured and was reduced 21% at week 3, 15% at week 6, 24% at week 12, 28% at week 18, and 31% at week 24 compared to week 0 (Figure 2C). The Cer d16:0 composed ~1% of the total ceramides and was significantly reduced ~46% at week 18 compared to week 0 (Table S3 in Supplementary Material). Ceramide 22:0 comprised ~7% of total ceramides and was significantly increased 96% at week 3, 59% at week 6, 69% at week 12, 44% at week 18, and 50% at week 24 compared to week 0 (Figure 2B). Ceramide 24:0 comprised roughly 11% of total ceramides and was significantly increased 183% at week 3, 98% at week 6, 129% at week 12, 66% at week 18, and 111% at week 24 compared to week 0 (Figure 2D). Ceramide 26:0 comprised ~1% of total ceramides and was significantly increased 143% at week 3, 85% at week 6, 149% at week 12, 71% at week 18, and 111% at week 24 compared to week 0 (Figure 2F). No statistically significant change in serum levels of Cer 14:0, Cer 16:0, Cer 18:0, Cer 20:0, Cer 22:1, and Cer 26:1, which comprised roughly 3, 14, 9, 3, 4, and 5% of total serum ceramides measured, respectively, were observed compared to week 0 (Table S3 in Supplementary Material).

### Serum Sphingosine Levels Increased on a Modified Diet

**Figure 3:**
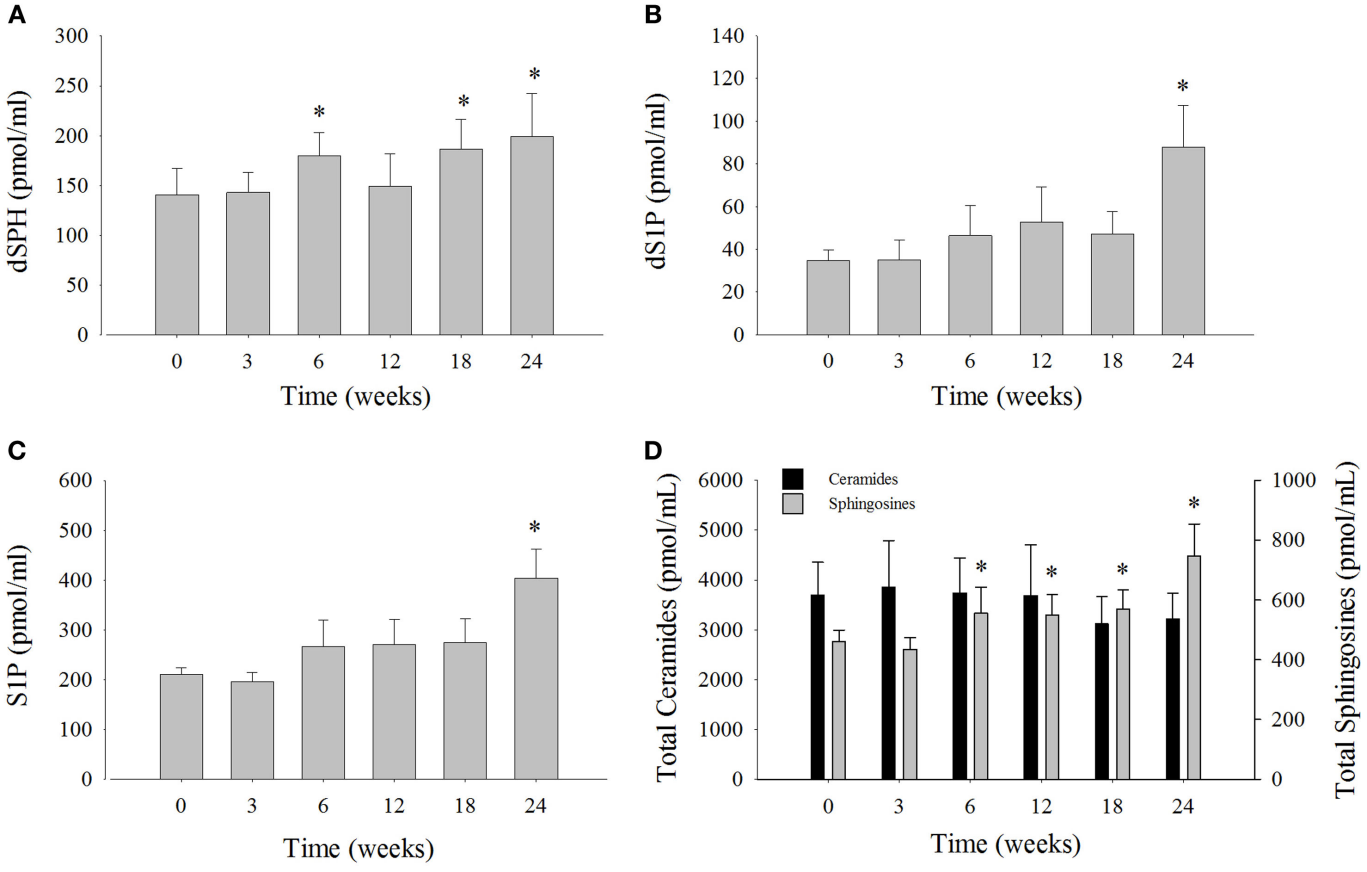
**Serum sphingolipid levels and total serum ceramide levels over the 24-week study from bottlenose dolphins on a modified fish diet**. Abundances of the sphingolipids **(A)** dihydrosphingosine (dSPH), **(B)** dihydrosphingosine 1-phosphoste (dS1P), and **(C)** sphingosine 1-phosphate (S1P) were significantly increased at week 24 compared to week 0. **(D)** Total sphingosines represented as the sum of dSPH, dS1P, sphingosine, and S1P were increased compared to week 0, whereas total ceramides represented as the sum of ceramides listed in Table S2 in Supplementary Material were not significantly different. A repeated measures one-way ANOVA with a Holm–Sidak *post hoc* test was used to determine significant changes in individual sphingosine levels versus week 0 (* denotes *P* < 0.05). Data are reported as mean ± SD.

Serum sphingosine levels were compared at each time point to week 0 (Figure 3; Table S3 in Supplementary Material). Dihydrosphingosine was significantly elevated by 32% at 6 weeks (180 ± 24 pmol/ml), 34% at 18 weeks (186 ± 30 pmol/ml), and 45% at 24 weeks (199 ± 43 pmol/ml) compared to week 0 (140 ± 27 pmol/ml). The levels of dS1P were significantly elevated by 157% at week 24 (88 ± 20 pmol/ml) compared to week 0 (35 ± 5 pmol/ml) (Figure 3B). The most abundant sphingosine measured was S1P that comprised ~49% of the total SPH and was significantly elevated by 92% at week 24 (404 ± 59 pmol/ ml) compared to week 0 (211 ± 13 pmol/ml) (Figure 3C). The levels of total SPH (sum of SPH measured) were significantly increased by 21% at 6 weeks, 19% at 12 weeks, 24% at 18 weeks, and 62% at 24 weeks after the change in diet (Figure 3D). Mean total ceramides were numerically lower, but were not statistically significant compared to time 0 (Figure 3D). The change in serum levels of SPH, which comprised roughly 12% of the total SPH, was not statistically significant compared to week 0 (Table S3 in Supplementary Material).

### Significant Changes in Serum Proteins Identified by Mass Spectrometry

Mass spectrometry-based proteomics of undepleted serum led to the identification of 59 proteins with a false discovery rate <0.1%. Eight proteins were significantly different over the 24-week study relative to time 0 (Table 2). Corroborating the PRM-MS data, adiponectin was significantly elevated by 2.66-, 2.79-, and 2.99-fold at weeks 12, 18, and 24 compared to week 0, respectively (Table 2). Haptoglobin was elevated by 1.72-fold at week 12 and 1.55-fold at week 18, compared to week 0 (Table 2). Inter-alpha (globulin) inhibitor H3 displayed a 1.51-fold increase only at week 12 compared to week 0 (Table 2). Serpin peptidase inhibitor, clade C-1 (antithrombin III) was the only protein significantly reduced, but this reduction was transient and only reduced at week 6 (−1.50-fold change) compared to week 0 (Table 2). Although ANOVA identified Hemoglobin subunit beta, hemoglobin subunit alpha, apolipoprotein E, and albumin as significantly different, *post hoc* analysis did not indicate statistical differences at any time point compared to week 0 (Table 2).

**Table 2:** **Changes in quantitative spectral counts normalized to total TIC for proteins identified in the serum of the 24-week feeding study dolphins that were significantly different over time**.

Identified proteins	Ensemble ID	RM-ANOVA (*P* < 0.05)	Fold change from week 0			
			0 versus 6	0 versus 12	0 versus 18	0 versus 24
SERPINC1 (antithrombin)	ENSTTRP00000008123	0.033	−1.50*	−1.07	1.08	−1.22
Hemoglobin subunit beta	ENSTTRP00000016564	0.03	−1.34	1.84	1.58	1.17
Haptoglobin	ENSTTRP00000001793	0.041	1.02	1.72*	1.55*	1.02
Hemoglobin subunit alpha	ENSTTRP00000011461	0.048	−1.29	1.89	1.63	1.14
Apolipoprotein E	ENSTTRP00000008256	0.041	−1.59	1.15	1.21	1.28
Adiponectin	ENSTTRP00000015964	0.001	1.93	2.66*	2.79*	2.99*
Albumin	ENSTTRP00000006225	0.049	1.02	−1.03	−1.01	1.02
Inter-α (globulin) inhibitor H3	ENSTTRP00000002122	0.021	−1.73	1.51*	1.24	1.33

*P < 0.05 for indicates a significant fold change using Holm–Sidak *Post hoc* test for multiple comparisons to control (week 0).

*Fold change was calculated using non-transformed means*.

## Discussion

Feeding dolphins a modified fish diet can reduce serum ferritin concentration within 3 weeks and normalize insulin, glucose, and triglycerides by 24 weeks (3). A modified diet with increased dietary intake of C17:0 has been proposed as a primary contributor to these effects. It was speculated that decreased ferritin, in part, may have led to an increase in insulin sensitivity. This speculation seems reasonable given that hyperferritinemia is a marker of insulin resistance (21) and that lowering serum ferritin is known to improve insulin resistance in humans with liver disease (22). The link between iron and the insulin sensitizing adipokine, adiponectin, has also been well established (23) where high serum iron leads to a reduction in serum adiponectin and in turn decreases insulin sensitivity.

Because of the challenges in determining insulin resistance in dolphins, we decided to investigate hormonal changes in the insulin sensitizing FGF21/adiponectin/ceramide axis that would be consistent with improving insulin sensitivity (5). The insulin sensitizing hormone adiponectin increased in dolphins fed a modified diet over the 24-week period, which supports the hypothesis of increasing insulin sensitivity in dolphins fed a diet higher in mullet or pinfish (3). Furthermore, there was a positive correlation between adiponectin and serum levels of the fatty acid C17:0, supporting the notion that elevated C17:0 may be a contributor to adiponectin mediated insulin sensitivity. There was also an inverse correlation between adiponectin and several serum iron indices; ferritin, total iron, and percent transferrin saturation (Table 1), suggesting that iron status is related to circulating adiponectin concentration in dolphins, as has been reported for humans (23). At the population level, total adiponectin is not different between dolphins with elevated iron versus healthy controls (17) which appears to contradict these findings. However, in this study we considered each individual as its own control and individual changes from baseline demonstrated a greater power to discover changes in adiponectin over time following intervention (Figure 1C).

Since high levels of serum ferritin are associated with lower HMW levels of adiponectin in people with type 2 diabetes (24), we measured percent unmodified adiponectin, which is considered a proxy of HMW adiponectin (17), and has been shown to discriminate between dolphins with high iron load versus healthy controls at a 2 h post-prandial time point. In non-diabetic humans, the post-prandial reduction in serum HMW adiponectin, but not total adiponectin, is considered a normal response (25); whereas, insulin-resistant humans do not exhibit a reduction in post-prandial serum HMW adiponectin. If insulin sensitivity was being restored in the study dolphins, then a reduction in percent unmodified adiponectin, i.e., HMW adiponectin, should be observed after feeding, like that shown for non-diabetic humans. As early as 3 weeks, percent unmodified adiponectin was reduced and remained reduced over the 24 week study (Figure 1D). The HMW adiponectin response to feeding in the study dolphins are consistent with the improved insulin sensitivity observed in non-diabetic humans (25). Furthermore, post-prandial percent unmodified adiponectin was also positively correlated with serum ferritin levels, indicating that improvement in the HMW adiponectin response could be due to lower serum ferritin or vice versa. We propose that the reduction in percent unmodified adiponectin in dolphins represents a picture similar to non-diabetic humans that supports the contention that insulin sensitivity is being restored in dolphins fed a modified diet that included increased C17:0 intake.

### FGF21–Adiponectin–Ceramide Axis

FGF21 expression has been shown to elicit a multitude of beneficial metabolic responses ranging from reducing blood glucose, improving lipid utilization, and elevating insulin sensitivity (26, 27). Many of the beneficial effects of FGF21 have been ascribed to its ability to induce adiponectin and lead to the sequential reduction in serum ceramides, also known as the FGF21/adiponectin/ceramide axis (5). Overexpression of FGF21 increases adiponectin and reduces ceramides in the serum of rodents, but is ineffective at lowering blood glucose and ceramide levels in adiponectin knockout mice (5, 28). To determine whether the elevation in total adiponectin in dolphins on a C17:0-rich diet was preceded by an increase in FGF21, we utilized a commercial ELISA previously used to measure serum FGF21 in cattle (29, 30). The serum FGF21 concentrations were highly variable among this study’s dolphin population (129–1599 pg/ml), but was within similar ranges reported for humans 21–5300 pg/ml (31), pregnant cows 15–1600 pg/ml (29), pigs 225–325 pg/ml (32), northern elephant seals ~100–500 pg/ml (33), and rhesus monkeys 10–325 pg/ml (34). Even though we observed no significant difference between FGF21 levels over time, FGF21 was highly correlated with the abundance of total adiponectin (Table 1). The tight correlation with adiponectin levels suggest that both hormones are linked, and the lack of changes in FGF21 concentration over time may be explained by its dynamic nature. In humans, post-prandial FGF21 levels decrease in a similar manner to HMW adiponectin with the highest relative reduction being greatest in healthy individuals (35, 36). As a consequence of not having fasted FGF21 levels, we are unable to determine if the relative reduction in 2 h post-prandial FGF21 levels improve on the diet. Given the tight correlation with adiponectin, it is possible that the FGF21/adiponectin axis is functional in dolphins. However, the lack of a clear elevation in FGF21 levels over the feeding study does not allow us to conclude that FGF21 plays a direct role in the elevation of adiponectin. A more intensive sampling regime at both fasting and post-prandial time points for each dolphin is required to test whether changes in FGF21 precedes changes in adiponectin.

The third arm of the FGF21 axis includes ceramides and SPH. Total serum ceramide levels are elevated in diabetic animals (37, 38) and humans, along with reduced total serum SPH (39). Activation of adiponectin receptors leads to the downstream activation of ceramidase that converts ceramides into SPH resulting in increased systemic levels of the pro-survival S1P (11). The increase in S1P likely conveys the pro-survival effects that adiponectin has demonstrated on pancreatic beta cells (40), as well as its preventative role in podocyte injury (41) and liver regeneration (42). The balance between ceramides to S1P in dolphins had not been previously described; however, our results support a role for adiponectin in this balancing act driving the scale toward S1P-mediated insulin sensitivity. Consistent with the FGF21/Adiponectin/ceramide axis (5), total serum sphingosine was elevated over time implicating an increase in ceramidase activity. The major dolphin serum ceramide (Cer 24:1, Figure S3 in Supplementary Material) was significantly reduced at 6 weeks and remained depressed through 24 weeks, even though total serum ceramides were not significantly decreased as would be expected if adiponectin was acting through ceramidase. Interestingly, Cer 24:1 did not inversely correlate with total adiponectin, but may be relevant to metabolic syndrome as it is one of the most elevated ceramides in the plasma of female children with type 2 diabetes (43). Of note, the unsaturated ceramides Cer 24:1, 20:1, and 18:1 were all reduced from 6 to 24 weeks, whereas the saturated ceramides Cer 26:0, 24:0, 22:0 were all elevated from 3 to 24 weeks. This result may be explained by the fact that unsaturated ceramides are the preferred substrate for alkaline ceramidases, which is preferentially enhanced upon stimulation of adiponectin receptors (11, 44). Feeding may also underlie some of these differences, in that, saturated ceramides Cer 20:0, Cer 18:0, and Cer 16:0 are higher in non-fasted humans compared to fasted humans (45), whereas fewer unsaturated ceramides were found to change with fasting. Because dolphins differ from humans in both total ceramides and ceramide composition (Figures S3 and S4 in Supplementary Material), it is difficult to extend findings of individual ceramide species in dolphins to specific clinical diseases in humans. However, the changes in the major ceramide species and total SPH are in agreement with that predicted if insulin sensitivity was being restored.

### Proteomics Results

To investigate whether changes in non-targeted serum proteins were being affected by dietary change, a cursory proteomic analysis of serum digests was conducted. Of the 59 high abundance proteins identified, eight proteins were considered statistically different. Adiponectin was found to be elevated at weeks 12–24, consistent with the targeted mass spectrometry assay data. Aside from adiponectin, only one other protein was elevated at more than one time point. Haptoglobin was elevated at weeks 12 and 18 and follows a pattern of abundance similar to that of the hemoglobin subunits. Haptoglobin binds to free hemoglobin to facilitate its removal from blood, which prevents the oxidative damage due to iron (46). Obese rats supplemented with *n*-3 polyunsaturated fatty acids showed a significant increase in post-prandial haptoglobin compared to control diet animals, although a mechanism underlying this elevation was not stated (47). The transient changes in serum haptoglobin could imply a small transient acute phase pro-inflammatory response, but this result could also be simply ascribed to different levels of hemolysis during the blood draw. Differences in haptoglobin levels were not previously observed for this study population determined by a standardized assay (3). Overall, changes in high abundance serum proteins were largely absent aside from adiponectin.

### Limitations

Because we were unable to obtain 2-h post-prandial serum samples from dolphins on a standard diet, we cannot determine whether or not the response in serum adiponectin, ceramides, or SPH are due to season. For example, managed spinner dolphins (*Stenella longirostris*) exhibit seasonal variations in testosterone concentration (48). Seasonal changes in blood chemistry values and the skin transcriptome have also been documented in bottlenose dolphins (49, 50). Although not overwhelmingly correlated, sex hormones have been weakly correlated with adiponectin levels in humans (51, 52). As a result, the fluctuation in adiponectin levels could be affected by seasonal changes over the 24-week period collected for this study. Therefore, caution should be taken when ascribing effects due to the modified diet alone. Lastly, the use of a pan-mammalian ELISA to measure FGF21 concentrations has not been validated for dolphin FGF21. The lack of a recombinant standard dolphin FGF21 protein did not allow for calculations of cross-reactivity.

## Conclusion

These data provide indirect support of improved insulin sensitivity in dolphins fed a modified diet, which included increased C17:0 intake, based on increasing adiponectin serum concentration and SPH. With the exception of FGF21, our data support the hypothesis that dietary intervention in dolphins leads to an elevation in total adiponectin, an elevation in total sphingosine, and a reduction in the major ceramide species (Cer 24:1). Significant correlations between serum iron, serum ferritin, FGF21, and adiponectin suggest that the FGF21/adiponectin/ceramide axis is a regulatory hormonal axis in dolphins.

## Author Contributions

PS conceptualized and performed the mass spectrometry experiments, analyzed all the data, and participated in writing the manuscript. TH performed and analyzed the ELISA results and participated in writing the manuscript. CP and SV-W conducted the animal experiments, provided the samples, and participated in writing the manuscript. MJ conceptualized and assisted with analyzing the experiments and participated in writing the manuscript.

## Conflict of Interest Statement

Scientific findings from the original published modified diet dolphin study (Venn-Watson et al., 2015) are part of pending United States (Serial Nos. 14/591660, 14/980167, 14/980304, 14/980695, 14/981130) and International (PCTUS1567172, G89743/194524) patents filed by the United States Navy. A small business, Epitracker, co-founded by the co-author (SV-W) has acquired an exclusive license from the U.S. Navy to practice these inventions.

## Abbreviations

Cer, ceramide; dS1P, dihydro-sphingosine-1-phosphates; dSPH, dihydro-sphingosines; FGF21, fibroblast growth factor 21; HMW, high molecular weight; S1P, sphingosine-1-phosphates; SPH, sphingosines.

## References

[1] Venn-Watson S Benham C Carlin K DeRienzo D St Leger J. Hemochromatosis and fatty liver disease: building evidence for insulin resistance in bottlenose dolphins (*Tursiops truncatus*). J Zoo Wildl Med (2012) 43:S35–47. 10.1638/2011-0146.1 23156704

[2] Venn-Watson S Smith CR Stevenson S Parry C Daniels R Jensen E Blood-based indicators of insulin resistance and metabolic syndrome in bottlenose dolphins (*Tursiops truncatus*). Front Endocrinol (2013) 4:136. 10.3389/fendo.2013.00136 24130551 PMC3793200

[3] Venn-Watson SK Parry C Baird M Stevenson S Carlin K Daniels R Increased dietary intake of saturated fatty acid heptadecanoic acid (C17:0) associated with decreasing ferritin and alleviated metabolic syndrome in dolphins. PLoS One (2015) 10:e0132117. 10.1371/journal.pone.0132117 26200116 PMC4511797

[4] Park SE Park CY Sweeney G. Biomarkers of insulin sensitivity and insulin resistance: past, present and future. Crit Rev Clin Lab Sci (2015) 52:180–90. 10.3109/10408363.2015.1023429 26042993

[5] Holland WL Adams AC Brozinick JT Bui HH Miyauchi Y Kusminski CM An FGF21-adiponectin-ceramide axis controls energy expenditure and insulin action in mice. Cell Metab (2013) 17:790–7. 10.1016/j.cmet.2013.03.019 23663742 PMC3667496

[6] Rutkowski JM Scherer PE. Isolation and quantitation of adiponectin higher order complexes. Methods Enzymol (2014) 537:243–59. 10.1016/B978-0-12-411619-1.00013-6 24480350 PMC4040967

[7] Neumeier M Weigert J Schaffler A Wehrwein G Muller-Ladner U Scholmerich J Different effects of adiponectin isoforms in human monocytic cells. J Leukoc Biol (2006) 79:803–8. 10.1189/jlb.0905521 16434692

[8] Miller RA Chu Q Le Lay J Scherer PE Ahima RS Kaestner KH Adiponectin suppresses gluconeogenic gene expression in mouse hepatocytes independent of LKB1-AMPK signaling. J Clin Invest (2011) 121:2518–28. 10.1172/JCI45942 21606593 PMC3104763

[9] Schulze MB Rimm EB Shai I Rifai N Hu FB. Relationship between adiponectin and glycemic control, blood lipids, and inflammatory markers in men with type 2 diabetes. Diabetes Care (2004) 27:1680–7. 10.2337/diacare.27.7.1680 15220246

[10] Mantzoros CS Li T Manson JE Meigs JB Hu FB. Circulating adiponectin levels are associated with better glycemic control, more favorable lipid profile, and reduced inflammation in women with type 2 diabetes. J Clin Endocrinol Metab (2005) 90:4542–8. 10.1210/jc.2005-0372 15914524

[11] Holland WL Miller RA Wang ZV Sun K Barth BM Bui HH Receptor-mediated activation of ceramidase activity initiates the pleiotropic actions of adiponectin. Nat Med (2011) 17:55–63. 10.1038/nm.2277 21186369 PMC3134999

[12] Hannun YA Obeid LM. Principles of bioactive lipid signalling: lessons from sphingolipids. Nat Rev Mol Cell Biol (2008) 9:139–50. 10.1038/nrm2329 18216770

[13] Holland WL Brozinick JT Wang LP Hawkins ED Sargent KM Liu Y Inhibition of ceramide synthesis ameliorates glucocorticoid-, saturated-fat-, and obesity-induced insulin resistance. Cell Metab (2007) 5:167–79. 10.1016/j.cmet.2007.01.002 17339025

[14] Lancaster GI Febbraio MA. Adiponectin sphings into action. Nat Med (2011) 17:37–8. 10.1038/nm0111-37 21217676

[15] Wang Y Lam KS Chan L Chan KW Lam JB Lam MC Post-translational modifications of the four conserved lysine residues within the collagenous domain of adiponectin are required for the formation of its high molecular weight oligomeric complex. J Biol Chem (2006) 281:16391–400. 10.1074/jbc.M513907200 16621799

[16] Wang Y Xu A Knight C Xu LY Cooper GJ. Hydroxylation and glycosylation of the four conserved lysine residues in the collagenous domain of adiponectin. Potential role in the modulation of its insulin-sensitizing activity. J Biol Chem (2002) 277:19521–9. 10.1074/jbc.M200601200 11912203

[17] Neely BA Carlin KP Arthur JM McFee WE Janech MG. Ratiometric measurements of adiponectin by mass spectrometry in bottlenose dolphins (*Tursiops truncatus*) with iron overload reveal an association with insulin resistance and glucagon. Front Endocrinol (2013) 4:132. 10.3389/fendo.2013.00132 24065958 PMC3778387

[18] Bielawski J Szulc ZM Hannun YA Bielawska A. Simultaneous quantitative analysis of bioactive sphingolipids by high-performance liquid chromatography-tandem mass spectrometry. Methods (2006) 39:82–91. 10.1016/j.ymeth.2006.05.004 16828308

[19] Lindblad-Toh K Garber M Zuk O Lin MF Parker BJ Washietl S A high-resolution map of human evolutionary constraint using 29 mammals. Nature (2011) 478:476–82. 10.1038/nature10530 21993624 PMC3207357

[20] Vizcaino JA Deutsch EW Wang R Csordas A Reisinger F Rios D ProteomeXchange provides globally coordinated proteomics data submission and dissemination. Nat Biotechnol (2014) 32:223–6. 10.1038/nbt.2839 24727771 PMC3986813

[21] Fernandez-Real JM Ricart-Engel W Arroyo E Balanca R Casamitjana-Abella R Cabrero D Serum ferritin as a component of the insulin resistance syndrome. Diabetes Care (1998) 21:62–8. 10.2337/diacare.21.1.62 9580307

[22] Valenti L Fracanzani AL Dongiovanni P Bugianesi E Marchesini G Manzini P Iron depletion by phlebotomy improves insulin resistance in patients with nonalcoholic fatty liver disease and hyperferritinemia: evidence from a case-control study. Am J Gastroenterol (2007) 102:1251–8. 10.1111/j.1572-0241.2007.01192.x 17391316

[23] Gabrielsen JS Gao Y Simcox JA Huang J Thorup D Jones D Adipocyte iron regulates adiponectin and insulin sensitivity. J Clin Invest (2012) 122:3529–40. 10.1172/JCI44421 22996660 PMC3461897

[24] Aso Y Takebayashi K Wakabayashi S Momobayashi A Sugawara N Terasawa T Relation between serum high molecular weight adiponectin and serum ferritin or prohepcidin in patients with type 2 diabetes. Diabetes Res Clin Pract (2010) 90:250–5. 10.1016/j.diabres.2010.09.008 20888657

[25] Ozeki N Hara K Yatsuka C Nakano T Matsumoto S Suetsugu M Serum high-molecular weight adiponectin decreases abruptly after an oral glucose load in subjects with normal glucose tolerance or impaired fasting glucose, but not those with impaired glucose tolerance or diabetes mellitus. Metabolism (2009) 58:1470–6. 10.1016/j.metabol.2009.04.042 19592051

[26] Itoh N. Hormone-like (endocrine) Fgfs: their evolutionary history and roles in development, metabolism, and disease. Cell Tissue Res (2010) 342:1–11. 10.1007/s00441-010-1024-2 20730630 PMC2948652

[27] Kharitonenkov A Larsen P. FGF21 reloaded: challenges of a rapidly growing field. Trends Endocrinol Metab (2011) 22:81–6. 10.1016/j.tem.2010.11.003 21194964

[28] Lin Z Tian H Lam KS Lin S Hoo RC Konishi M Adiponectin mediates the metabolic effects of FGF21 on glucose homeostasis and insulin sensitivity in mice. Cell Metab (2013) 17:779–89. 10.1016/j.cmet.2013.04.005 23663741

[29] Schoenberg KM Giesy SL Harvatine KJ Waldron MR Cheng C Kharitonenkov A Plasma FGF21 is elevated by the intense lipid mobilization of lactation. Endocrinology (2011) 152:4652–61. 10.1210/en.2011-1425 21990311

[30] Akbar H Batistel F Drackley JK Loor JJ. Alterations in hepatic FGF21, co-regulated genes, and upstream metabolic genes in response to nutrition, ketosis and inflammation in peripartal Holstein cows. PLoS One (2015) 10:e0139963. 10.1371/journal.pone.0139963 26451842 PMC4599736

[31] Galman C Lundasen T Kharitonenkov A Bina HA Eriksson M Hafstrom I The circulating metabolic regulator FGF21 is induced by prolonged fasting and PPARalpha activation in man. Cell Metab (2008) 8:169–74. 10.1016/j.cmet.2008.06.014 18680716

[32] Varady J Ringseis R Eder K. Dietary moderately oxidized oil induces expression of fibroblast growth factor 21 in the liver of pigs. Lipids Health Dis (2012) 11:34. 10.1186/1476-511X-11-34 22394566 PMC3807756

[33] Suzuki M Lee AY Vazquez-Medina JP Viscarra JA Crocker DE Ortiz RM Plasma FGF21 concentrations, adipose fibroblast growth factor receptor-1 and beta-klotho expression decrease with fasting in northern elephant seals. Gen Comp Endocrinol (2015) 216:86–9. 10.1016/j.ygcen.2015.03.009 25857751 PMC4457680

[34] Nygaard EB Moller CL Kievit P Grove KL Andersen B. Increased fibroblast growth factor 21 expression in high-fat diet-sensitive non-human primates (*Macaca mulatta*). Int J Obes (Lond) (2014) 38:183–91. 10.1038/ijo.2013.79 23736354 PMC4376022

[35] Matikainen N Taskinen MR Stennabb S Lundbom N Hakkarainen A Vaaralahti K Decrease in circulating fibroblast growth factor 21 after an oral fat load is related to postprandial triglyceride-rich lipoproteins and liver fat. Eur J Endocrinol (2012) 166:487–92. 10.1530/EJE-11-0783 22190000

[36] Reinhard M Frystyk J Jespersen B Randers E Bibby BM Ivarsen P. Response of fibroblast growth factor 21 to meal intake and insulin infusion in patients on maintenance haemodialysis. Clin Endocrinol (Oxf) (2015) 83:187–95. 10.1111/cen.12737 25659979

[37] Samad F Hester KD Yang G Hannun YA Bielawski J. Altered adipose and plasma sphingolipid metabolism in obesity: a potential mechanism for cardiovascular and metabolic risk. Diabetes (2006) 55:2579–87. 10.2337/db06-0330 16936207

[38] Brozinick JT Hawkins E Hoang Bui H Kuo MS Tan B Kievit P Plasma sphingolipids are biomarkers of metabolic syndrome in non-human primates maintained on a Western-style diet. Int J Obes (Lond) (2013) 37:1064–70. 10.1038/ijo.2012.191 23207405 PMC3718866

[39] Bergman BC Brozinick JT Strauss A Bacon S Kerege A Bui HH Serum sphingolipids: relationships to insulin sensitivity and changes with exercise in humans. Am J Physiol Endocrinol Metab (2015) 309:E398–408. 10.1152/ajpendo.00134.2015 26126684 PMC4537923

[40] Rakatzi I Mueller H Ritzeler O Tennagels N Eckel J. Adiponectin counteracts cytokine- and fatty acid-induced apoptosis in the pancreatic beta-cell line INS-1. Diabetologia (2004) 47:249–58. 10.1007/s00125-003-1293-3 14722646

[41] Rutkowski JM Wang ZV Park AS Zhang J Zhang D Hu MC Adiponectin promotes functional recovery after podocyte ablation. J Am Soc Nephrol (2013) 24:268–82. 10.1681/ASN.2012040414 23334396 PMC3559480

[42] Ezaki H Yoshida Y Saji Y Takemura T Fukushima J Matsumoto H Delayed liver regeneration after partial hepatectomy in adiponectin knockout mice. Biochem Biophys Res Commun (2009) 378:68–72. 10.1016/j.bbrc.2008.10.176 19013135

[43] Lopez X Goldfine AB Holland WL Gordillo R Scherer PE. Plasma ceramides are elevated in female children and adolescents with type 2 diabetes. J Pediatr Endocrinol Metab (2013) 26:995–8. 10.1515/jpem-2012-0407 23612696

[44] Mao C Obeid LM. Ceramidases: regulators of cellular responses mediated by ceramide, sphingosine, and sphingosine-1-phosphate. Biochim Biophys Acta (2008) 1781:424–34. 10.1016/j.bbalip.2008.06.002 18619555 PMC2614331

[45] Hammad SM Pierce JS Soodavar F Smith KJ Al Gadban MM Rembiesa B Blood sphingolipidomics in healthy humans: impact of sample collection methodology. J Lipid Res (2010) 51:3074–87. 10.1194/jlr.D008532 20660127 PMC2936747

[46] Schaer DJ Vinchi F Ingoglia G Tolosano E Buehler PW. Haptoglobin, hemopexin, and related defense pathways-basic science, clinical perspectives, and drug development. Front Physiol (2014) 5:415. 10.3389/fphys.2014.00415 25389409 PMC4211382

[47] Hassanali Z Ametaj BN Field CJ Proctor SD Vine DF. Dietary supplementation of n-3 PUFA reduces weight gain and improves postprandial lipaemia and the associated inflammatory response in the obese JCR:LA-cp rat. Diabetes Obes Metab (2010) 12:139–47. 10.1111/j.1463-1326.2009.01130.x 19917068

[48] Wells R Reproductive behavior and hormonal correlates in Hawaiian spinner dolphins, *Stenella longirostris*. Reproduction on Whales, Dolphins, and Porpoises. Cambridge: Reports of the International Whaling Commission (1984). p. 465–72.

[49] Hall AJ Wells RS Sweeney JC Townsend FI Balmer BC Hohn AA Annual, seasonal and individual variation in hematology and clinical blood chemistry profiles in bottlenose dolphins (*Tursiops truncatus*) from Sarasota Bay, Florida. Comp Biochem Physiol A Mol Integr Physiol (2007) 148:266–77. 10.1016/j.cbpa.2007.04.017 17524692

[50] Van Dolah FM Neely MG McGeorge LE Balmer BC Ylitalo GM Zolman ES Seasonal variation in the skin transcriptome of common bottlenose dolphins (*Tursiops truncatus*) from the northern Gulf of Mexico. PLoS One (2015) 10:e0130934. 10.1371/journal.pone.0130934 26110790 PMC4482424

[51] Tworoger SS Mantzoros C Hankinson SE. Relationship of plasma adiponectin with sex hormone and insulin-like growth factor levels. Obesity (Silver Spring) (2007) 15:2217–24. 10.1038/oby.2007.263 17890489

[52] Bai J Liu Y Niu GF Bai LX Xu XY Zhang GZ Relationship between adiponectin and testosterone in patients with type 2 diabetes. Biochem Med (Zagreb) (2011) 21:65–70. 10.11613/BM.2011.013 22141209

