# Supplementary material for: Feeding a Modified Fish Diet to Bottlenose Dolphins Leads to an Increase in Serum Adiponectin and Sphingolipids
Source: Front Endocrinol (Lausanne). 2016 Apr 21;7:33. doi: 10.3389/fendo.2016.00033 (PMC4838613; doi:10.3389/fendo.2016.00033)
Supplement: Supplementary file 1 [file Table_1.DOCX]

Supplementary Material

**Feeding a Modified Fish Diet to Bottlenose Dolphins Leads to an Increase in Serum Adiponectin and Sphingolipids**

**Philip M. Sobolesky^1^, Tyler Harrell^2^, Celeste Parry^3^, Stephanie Venn-Watson^3^ and Michael G. Janech^1,2*^**

^1^ Division of Nephrology, Department of Medicine, Medical University of South Carolina, Charleston, SC, USA.

^2^Grice Marine Laboratory, Department of Biology, College of Charleston, Charleston, SC, USA.

^3^Translational Medicine and Research Program, National Marine Mammal Foundation, San Diego, CA, USA.

***Correspondence:** Michael G. Janech, Division of Nephrology, Department of Medicine, Medical University of South Carolina, 114 Doughty Street STB443, Charleston, SC, USA.

janechmg@musc.edu

| **Supplemental Table 1 – Comparisons of Dietary Nutrients in Base versus Modified Diet for Six Bottlenose Dolphins (*Tursiops Truncatus*)** | | | |
| --- | --- | --- | --- |
| **Dietary Variable**  **(calculated as daily intake per kg body weight)** | **Base Diet**  **(per kg body weight)** | **Modified Diet**  **(per kg body weight)** | **P value*** |
| Kilocalories | 54 ± 16 | 54 ± 17 | 0.50 |
| Moisture (g) | 32 ± 13 | 32 ± 12 | 0.50 |
| Protein (g) | 6 ± 2 | 7 ±2 | 0.34 |
| Fat (g) | 2 ± 1 | 2 ± 1 | 0.41 |
| Ash (g) | 0.6 ± 0.2 | 0.6 ± 0.2 | 0.50 |
| Carbohydrates (g) | 0.2 ± 0.1 | 0.3 ± 0.1 | 0.10 |
| *Wilcoxon two-sample test |  |  |  |
|  |  |  |  |
