# Supplementary material for: Feeding a Modified Fish Diet to Bottlenose Dolphins Leads to an Increase in Serum Adiponectin and Sphingolipids
Source: Front Endocrinol (Lausanne). 2016 Apr 21;7:33. doi: 10.3389/fendo.2016.00033 (PMC4838613; doi:10.3389/fendo.2016.00033)
Supplement: Supplementary file 2 [file Table_2.DOCX]

Supplementary Material

**Feeding a Modified Fish Diet to Bottlenose Dolphins Leads to an Increase in Serum Adiponectin and Sphingolipids**

**Philip M. Sobolesky^1^, Tyler Harrell^2^, Celeste Parry^3^, Stephanie Venn-Watson^3^ and Michael G. Janech^1,2*^**

^1^ Division of Nephrology, Department of Medicine, Medical University of South Carolina, Charleston, SC, USA.

^2^Grice Marine Laboratory, Department of Biology, College of Charleston, Charleston, SC, USA.

^3^Translational Medicine and Research Program, National Marine Mammal Foundation, San Diego, CA, USA.

***Correspondence:** Michael G. Janech, Division of Nephrology, Department of Medicine, Medical University of South Carolina, 114 Doughty Street STB443, Charleston, SC, USA.

janechmg@musc.edu

| **Supplemental Table 2 - Pearson product moment correlations (ρ) of Adiponectin, % Unmodified Adiponectin, and FGF21 with ceramides and blood laboratory measurements** | | | | | | |
| --- | --- | --- | --- | --- | --- | --- |
|  |  |  |  |  |  |  |
| **Sphingolipids** | Adiponectin^†^ (ρ) | *P*- value | % Unmodified Adiponectin^†^ (ρ) | *P*- value | FGF21^‡^ (ρ) | *P*- value |
| Cer 16:0 | -0.221 | 0.202 | 0.024 | 0.892 | -0.260 | 0.144 |
| Cer 22:0 | -0.089 | 0.611 | -0.251 | 0.146 | -0.203 | 0.258 |
| Cer 24:0 | 0.092 | 0.598 | -0.192 | 0.269 | 0.002 | 0.993 |
| Cer 24:1 | -0.263 | 0.126 | 0.195 | 0.261 | -0.124 | 0.493 |
| Cer 26:0 | 0.053 | 0.763 | -0.150 | 0.389 | -0.059 | 0.746 |
| Cer 26:1 | -0.027 | 0.879 | 0.222 | 0.724 | 0.045 | 0.804 |
| Total Ceramides | -0.286 | 0.095 | -0.005 | 0.979 | -0.232 | 0.195 |
| dhS1P | 0.073 | 0.677 | -0.284 | 0.099 | -0.203 | 0.256 |
| SPH | -0.022 | 0.901 | -0.028 | 0.873 | -0.206 | 0.251 |
|  |  |  |  |  |  |  |
| **Values from Venn-Watson *et al.* 2015** |  |  |  |  |  |  |
| Glucose | -0.059 | 0.735 | -0.103 | 0.557 | 0.053 | 0.768 |
| Triglycerides | -0.102 | 0.558 | 0.161 | 0.356 | -0.097 | 0.590 |
| Ceruloplasmin | -0.320 | 0.061 | -0.164 | 0.348 | -0.205 | 0.252 |
| Haptoglobin | 0.074 | 0.672 | -0.079 | 0.651 | -0.062 | 0.731 |
| ^†^n=35; ^‡^n=33 |  |  |  |  |  |  |
