# Supplementary material for: Feeding a Modified Fish Diet to Bottlenose Dolphins Leads to an Increase in Serum Adiponectin and Sphingolipids
Source: Front Endocrinol (Lausanne). 2016 Apr 21;7:33. doi: 10.3389/fendo.2016.00033 (PMC4838613; doi:10.3389/fendo.2016.00033)
Supplement: Supplementary file 3 [file Table_3.DOCX]

Supplementary Material

**Feeding a Modified Fish Diet to Bottlenose Dolphins Leads to an Increase in Serum Adiponectin and Sphingolipid**

**Philip M. Sobolesky^1^, Tyler Harrell^2^, Celeste Parry^3^, Stephanie Venn-Watson^3^ and Michael G. Janech^1,2*^**

^1^ Division of Nephrology, Department of Medicine, Medical University of South Carolina, Charleston, SC, USA.

^2^Grice Marine Laboratory, Department of Biology, College of Charleston, Charleston, SC, USA.

^3^Translational Medicine and Research Program, National Marine Mammal Foundation, San Diego, CA, USA.

***Correspondence:** Michael G. Janech, Division of Nephrology, Department of Medicine, Medical University of South Carolina, 114 Doughty Street STB443, Charleston, SC, USA.

janechmg@musc.edu

| **Supplemental Table 3. Comprehensive list of serum Ceramide and Sphingosine concentrations within the 24-week feeding study. Significant changes were indicated *versus* week 0.** | | | | | | |
| --- | --- | --- | --- | --- | --- | --- |
| **Serum Ceramides (pmols/ml ± SD)** | **Week 0** | **Week 3** | **Week 6** | **Week 12** | **Week 18** | **Week 24** |
| Cer 14:0 | 76 ± 19 | 94 ± 34 | 85 ± 20 | 102 ± 38 | 74 ± 25 | 81 ± 23 |
| Cer 16:0 | 435 ± 142 | 497 ±112 | 562 ± 180 | 518 ± 151 | 454 ± 103 | 398 ± 105 |
| Cer d16:0 | 70 ± 25 | 72 ± 49 | 49 ± 18 | 55 ± 23 | 36 ± 16^†^ | 46 ± 15 |
| Cer 18 | 316 ± 78 | 316 ±100 | 351 ± 84 | 363 ± 125 | 315 ± 63 | 276 ± 52 |
| Cer 18:1 | 78 ± 17 | 60 ± 12^†^ | 62 ± 12^†^ | 58 ± 17^†^ | 48 ± 14^‡^ | 56 ± 9^‡^ |
| Cer 20:0 | 133 ± 50 | 113 ± 29 | 130 ± 38 | 126 ± 45 | 123 ± 38 | 106 ± 33 |
| Cer 20:1 | 40 ± 13 | 31 ± 10^†^ | 33 ± 8^*^ | 30 ± 11^†^ | 28 ± 9^‡^ | 27 ± 9^‡^ |
| Cer 22:0 | 160 ± 50 | 259 ± 60^‡^ | 237 ± 49^†^ | 255 ± 60^‡^ | 215 ± 43^*^ | 225 ± 42^*^ |
| Cer 22:1 | 167 ± 23 | 168 ± 48 | 170 ± 38 | 157 ± 39 | 134 ± 23 | 129 ± 35 |
| Cer 24:0 | 187 ± 50 | 429 ± 171^‡^ | 350 ± 66^‡^ | 409 ± 121^‡^ | 290 ± 62^†^ | 378 ± 99^‡^ |
| Cer 24:1 | 1826 ± 289 | 1567 ± 338 | 1485 ± 280 | 1383 ± 406^*^ | 1223 ± 227^†^ | 1287 ± 205^†^ |
| Cer 26:0 | 16 ± 5 | 33 ± 12^†^ | 27 ± 8^*^ | 36 ± 20^†^ | 26 ± 11^*^ | 31 ± 14^*^ |
| Cer 26:1 | 186 ± 44 | 213 ± 89 | 194 ± 76 | 194 ± 88 | 144 ± 43 | 171 ± 52 |
| Total Ceramides | 3688 ± 672 | 3852 ± 933 | 3734 ± 699 | 3685 ± 1016 | 3112 ± 558 | 3213 ± 522 |
|  |  |  |  |  |  |  |
| **Serum Sphingosines (pmols/ml ± SD)** |  |  |  |  |  |  |
| dSPH | 140 ± 27 | 143 ± 20 | 180 ± 24^*^ | 149 ± 33 | 186 ± 30^*^ | 199 ± 43^†^ |
| dS1P | 35 ± 5 | 35 ± 9 | 46 ± 14 | 53 ± 16 | 47 ± 11 | 88 ± 20^‡^ |
| SPH | 74 ± 22 | 60 ± 19 | 63 ± 25 | 77 ± 43 | 61 ± 34 | 56 ± 7 |
| S1P | 211 ± 13 | 196 ± 19 | 266 ± 54 | 271 ± 50 | 275 ± 46 | 404 ± 59^‡^ |
| Total Sphingosines | 460 ± 39 | 434 ± 39 | 555 ± 86^*^ | 549 ± 69^*^ | 569 ± 64^†^ | 747 ± 107^‡^ |
| Significance was determined by a repeated measures one-way ANOVA with a Holm-Sidak post-hoc test.  * = *P <* 0.05, † = *P <* 0.01, and ‡ = *P* < 0.001. | | | | | | |
