# Supplementary material for: Feeding a Modified Fish Diet to Bottlenose Dolphins Leads to an Increase in Serum Adiponectin and Sphingolipids
Source: Front Endocrinol (Lausanne). 2016 Apr 21;7:33. doi: 10.3389/fendo.2016.00033 (PMC4838613; doi:10.3389/fendo.2016.00033)
Supplement: Supplementary file 4 [file Data_Sheet_1.DOCX]

Supplementary Material

**Feeding a Modified Fish Diet to Bottlenose Dolphins Leads to an Increase in Serum Adiponectin and Sphingolipids**

**Philip M. Sobolesky^1^, Tyler Harrell^2^, Celeste Parry^3^, Stephanie Venn-Watson^3^ and Michael G. Janech^1,2*^**

^1^ Division of Nephrology, Department of Medicine, Medical University of South Carolina, Charleston, SC, USA.

^2^Grice Marine Laboratory, Department of Biology, College of Charleston, Charleston, SC, USA.

^3^Translational Medicine and Research Program, National Marine Mammal Foundation, San Diego, CA, USA.

***Correspondence:** Michael G. Janech, Division of Nephrology, Department of Medicine, Medical University of South Carolina, 114 Doughty Street STB443, Charleston, SC, USA.

janechmg@musc.edu

Serum FGF21 concentrations were determined using the Fibroblast Growth Factor 21 Mouse/Rat ELISA kit (Biovendor, Asheville, NC). All solutions used in this procedure were provided by the manufacturer. Samples were thawed on ice for one hour, vortexed, then diluted 1:4 in dilution buffer. Standards were prepared by reconstituting the dry FGF21 standard in 1 mL of dilution buffer to a final concentration of 2560 pg/mL FGF21. Stock standard was serially diluted 1:1 down to 20 pg/mL resulting in 7 standard concentrations of: 1280, 640, 320, 160, 80, 40, and 20 pg/mL. Samples at time 0 from animal A, W, and LL were pooled in order to construct a standard reference material for estimation of batch variability. A volume of sample, standard, or standard reference material equal to 100µl was aliquoted in triplicate into the wells of two 96 well plates precoated with FGF21 antibody. Both samples and standards were randomized in location on the two plates. Triplicate samples from a single animal were always located on the same plate. Each plate contained an independent series of standards and standard reference material. Plates were incubated at room temperature (20°C) and shaken at 300 RPM for 1.6 hours. Plates were then washed three times with 35 µL of wash solution using a multi-channel pipette. Prior to and between washes the plates were inverted and tapped dry on a paper towel. Biotin Labelled Antibody solution (100 µL) was added to each well. Plates were further incubated at room temperature (20°C) and shaken at 300RPM for 1 hour. Plates were washed again as described above. Streptavidin-HRP Conjugate solution (100 µL) was then added to each well. Plates were incubated at room temperature (20°C) and shaken at 300RPM for 30 minutes and washed as described above. The substrate solution (100 µL) was added to each well. The plates were then covered with aluminum foil and incubated at room temperature (20°C) for 20 minutes. Color development was stopped by adding 100 µL of stop solution to each well. Absorbances were determined on a Spectramax 340PC microplate reader (Molecular Devices, Sunnyvale, CA) at 450 nm with a reference wavelength of 630 nm within 5 minutes of stop solution administration. Reference wavelength absorbance was subtracted from readings at 450 nm in order to obtain a final absorbance reading. A standard curve was generated using quadratic regression between standard absorbance’s and known concentrations. Lower limit of detection (LLOD) was determined by adding 3x the standard deviation (SD) of the blank to the mean of the blank. Lower limit of quantification (LLOQ) was determined by adding 10x the standard deviation of the blank to the mean of the blank. Batch corrections were not applied as the coefficient of variation for the mean of the standard reference material was 0.23%.
