# Supplementary figures and images for: Feeding a Modified Fish Diet to Bottlenose Dolphins Leads to an Increase in Serum Adiponectin and Sphingolipids
Source: Front Endocrinol (Lausanne). 2016 Apr 21;7:33. doi: 10.3389/fendo.2016.00033 (PMC4838613; doi:10.3389/fendo.2016.00033)

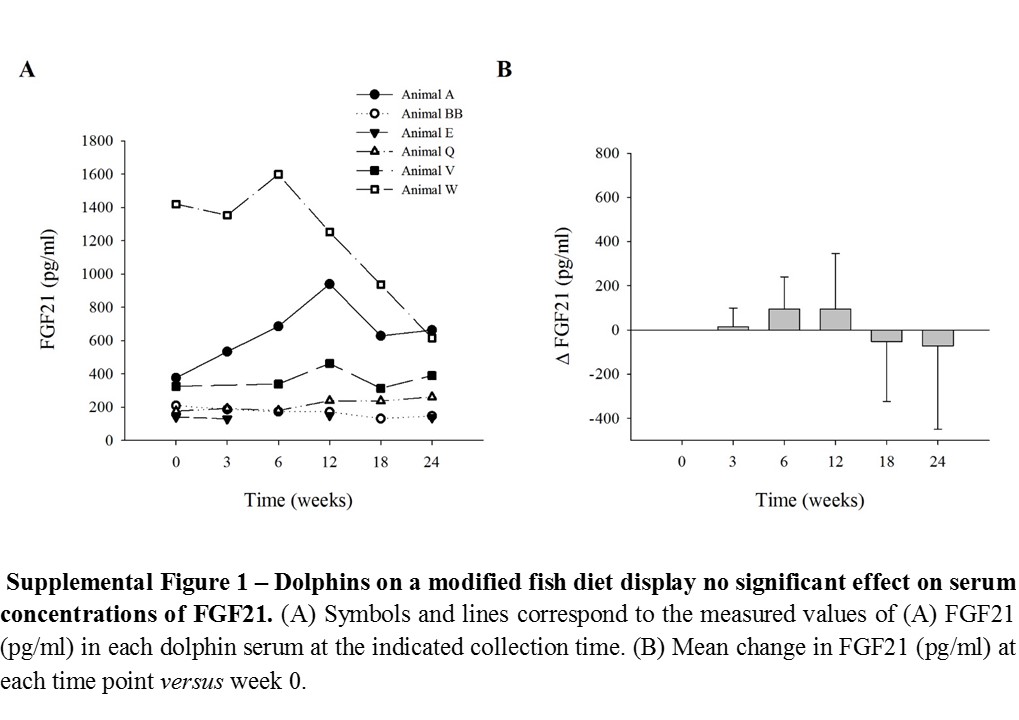

Supplement: Supplementary file 5 [file Image_1.JPEG]

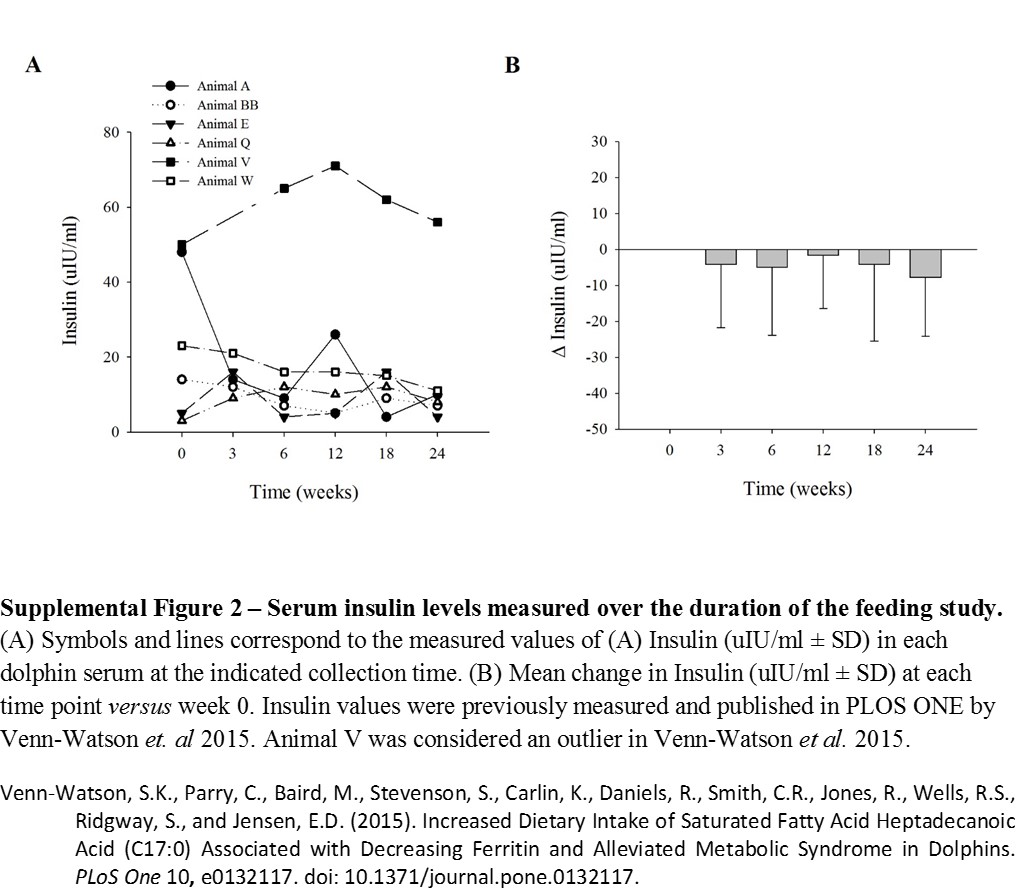

Supplement: Supplementary file 6 [file Image_2.JPEG]

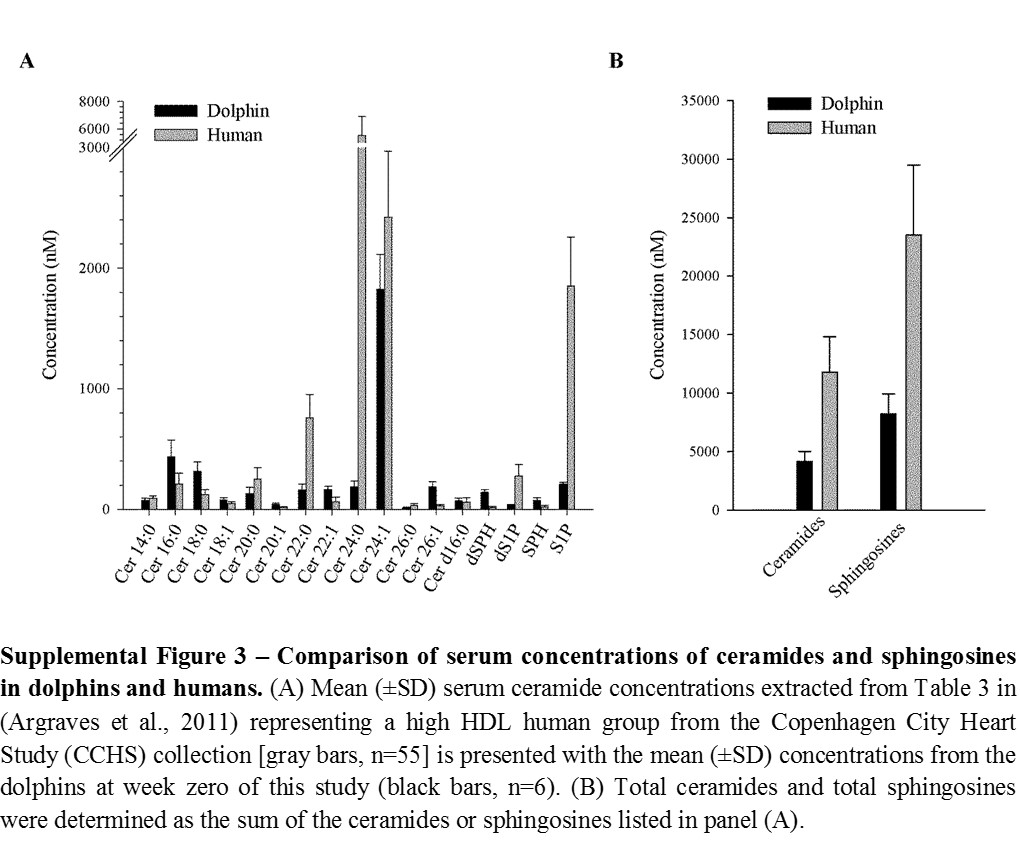

Supplement: Supplementary file 7 [file Image_3.JPEG]

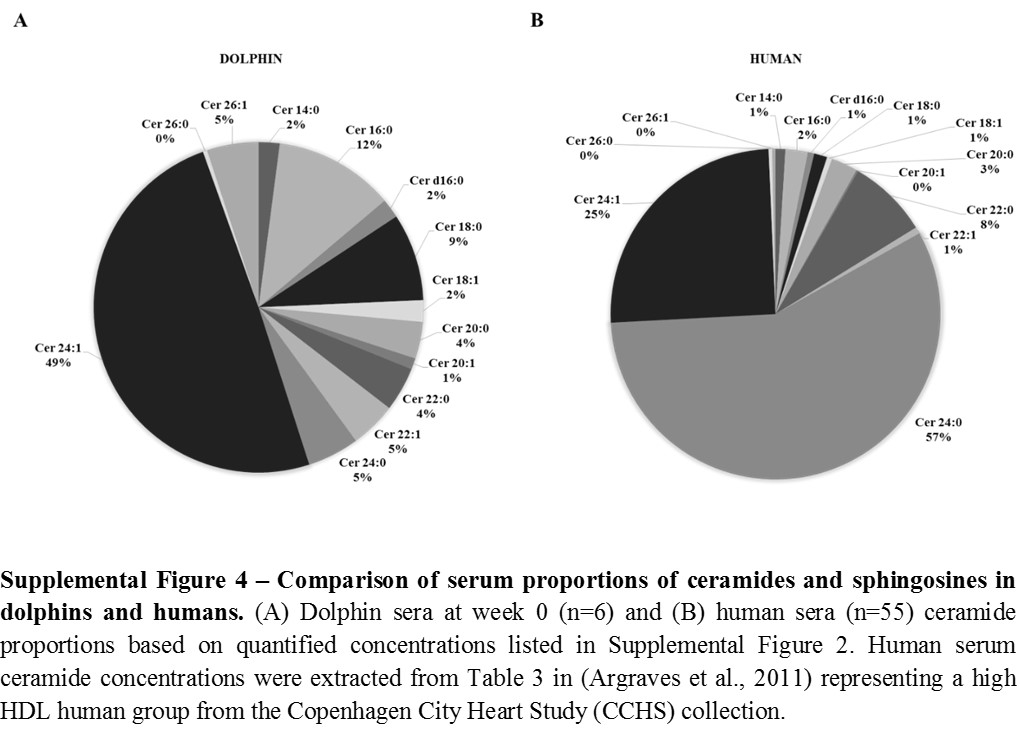

Supplement: Supplementary file 8 [file Image_4.JPEG]
